# Supplementary material for: Novel metabolic role for BDNF in pancreatic β-cell insulin secretion
Source: Nat Commun. 2020 Apr 23;11:1950. doi: 10.1038/s41467-020-15833-5 (PMC7181656; doi:10.1038/s41467-020-15833-5)
Supplement: Supplementary file 1 — Supplementary Information [file 41467_2020_15833_MOESM1_ESM.pdf]

Supplementary Information

Novel metabolic role for BDNF in pancreatic β-cell insulin secretion

Gianluca Fulgenzi[1^], Zhenyi Hong[1^], Francesco Tomassoni-Ardori[1], Luiz F. Barella[2], Jodi Becker[1], Colleen Barrick[1], Deborah Swing[1], Sudhirkumar Yanpallewar[1], Brad St Croix[1], Jürgen Wess[2], Oksana Gavrilova[3] and Lino Tessarollo[1*].

[1]Mouse Cancer Genetics Program, CCR, NCI, NIH; [2] Molecular Signaling Section, Laboratory of Bioorganic Chemistry and [3]Mouse Metabolism Core NIDDK, NIH;

^Equal Contribution

*Corresponding author: Lino Tessarollo Ph.D., NCI-Frederick, 7th Street, Bldg. 560, Frederick, MD, 21702, USA.

Email: tessarol@mail.nih.gov

Supplementary Table 1-3

Supplementary Figures 1-9

Supplementary Table 1

Clinical information of non-diabetic pancreatic islet donors.

Donor	Age	Race	Gender	BMI	HbA1c	Islet viability, purity	Cause of death
1	25	Hispanic	Male	27.7	5.2%	90%, 95%	Anoxia
2	44	White	Female	23.0	6.2%	96%, 80%	Cerebrovascular/stroke
3	52	Hispanic	Male	27.2	5.7%	96%, 85%	Cerebrovascular/stroke
4	57	Hispanic	Male	35.9	5.4%	95%, 80%	Cerebrovascular/stroke
5	51	White	Male	24.0	4.7%	95%, 80%	Head trauma

Supplementary Table 2

List of primers used for PCR studies (mouse)

Gene Name	Isoform	Primer Sequence (From 5' to 3')
Ntrk1 (TrkA, Mouse)		Forward: GCATGTCAACAACGGGAACT<br>Reverse: GAGAAGGAGACAGGGATGGG
Ntrk2 (TrkB, Mouse)	TrkB.FL	Common Forward: AGCAATCGGGAGCATCTCT<br>Reverse: CTGGCAGAGTCATCGTCGT
	TrkB.T1	Common Forward: AGCAATCGGGAGCATCTCT<br>Reverse: TACCCATCCAGTGGGATCTT
	TrkB.T2	Common Forward: AGCAATCGGGAGCATCTCT<br>Reverse: TCATGAGCCAAAAATGAGTCC
Ntrk3 (TrkC, Mouse)	TrkC.FL	Common Forward: TCCCAGTCATTGAGAACCCC<br>Reverse: CATCTTGTCTTTGGTGGGGC
	TrkC.T1	Common Forward: TCCCAGTCATTGAGAACCCC<br>Reverse: GGTAAGACACATCCCCACTCTG
Bdnf (Mouse)		Forward: CGACATCACTGGCTGACACT<br>Reverse: CAAAGGCACTTGACTGCTGA
Ntf5 (Mouse)		Forward: TGGCTCATCAAAACGGACGA<br>Reverse: CAGATGAGTACCACCGTGCA
Gapdh (Mouse)		Forward: TGCGACTTCAACAGCAACTC<br>Reverse: ATGTAGGCCATGAGGTCCAC
β-actin (Mouse)		Forward: CTATTGGCAACGAGCGGTTCC<br>Reverse: CAGCACTGTGTTGGCATAGAGG

List of primers used for PCR studies (human)

NTRK2 (TrkB, Human)	TrkB.FL	Common Forward: ATAAAACCGGTCGGGAACA<br>Reverse: TTGGAGATGTGATGGAGTGG
	TrkB.T1	Common Forward: ATAAAACCGGTCGGGAACA<br>Reverse: TACCCATCCAGTGGGATCTT
BDNF (Human)		Forward: AGCTGAGCGTGTGTGACAGT<br>Reverse: ATGGGATTGCACTTGGTCTC
NTF4 (Human)		Forward: GAATTGACACTGCCTGCGTCT<br>Reverse: TCCTTAGATCAGCTGGGCCAT
GAPDH (Human)		Forward: CACCAGGGCTGCTTTTAACT<br>Reverse: TGGGATTTCCATTGATGACA

Supplementary Table 3

List of antibodies used in this study

Antibody Name	Company and Catalog No.	Dilution	Application
TrkB	Millipore, 07-225	1:1000	Western blots
TrkB	R&D Systems, AF1494	1:50	Immunoelectron microscopy
		2 µg in 40 µl beads / sample	Immunoprecipitation
V5-Tag	Cell Signaling Technology, 13202	1:200	Immunostaining (Fig.1) IHC (Fig 4)
		1:1000	Western blots
Insulin	Cell Signaling Technology, 8138 (mouse)	1:800	Immunostaining (Fig.1 and SFig. 2))
Glucagon	Santa-Cruz, SC-7780 (goat)	1:200	Immunostaining (Fig.1)
Somatostatin	Santa-Cruz, SC-55565 (mouse)	1:200	Immunostaining (SFig.3)
CD31	Santa Cruz, SC-18916 (rat)	1:100	Immunostaining (Fig.1)
Insulin	Santa-Cruz, SC-7839 (goat)	1:200	Immunostaining (SFig.3)
Glucagon	Santa-Cruz, SC-13091 (rabbit)	1:200	Immunostaining (SFig.3)
FoxO1	Cell Signaling Technology, 2880	1:100	Immunostaining (SFig.3)
Desmin	Cell Signaling #D93F5	1:200	Immunostaining (Fig 4)
Dystrophin	Leica NCL-DYS-1	1:500	Immunostaining (Fig 6)
BDNF	AbCam #ab108319	1:1000	Western blot
TrkB-Fc	R&D Systems, 688-TK-100	1:100	Blocking BDNF in the diaphragm supernatant
β-actin	Santa-Cruz, SC-47778	1:3000	Western blot
GAPDH	MilliporeSigma, MAB374	1:3000	Western blot

List of antibodies used in this study (continued)

Antibody Name	Company and Catalog No.	Dilution	Application
p-ERK	Cell Signaling Technology, 9106	1:2000	Western blot
ERK	Cell Signaling Technology, 9102	1:2000	Western blot
6 nm colloidal gold donkey anti goat	Jackson ImmunoResearch, 705-195-147	1:200	Immunoelectron microscopy
Alexa Fluor 488 Donkey anti mouse	Thermo Fisher Scientific, A-21202	1:200	Immunofluorescence
Alexa Fluor 488 Donkey anti rabbit	Thermo Fisher Scientific, A-21206	1:200	Immunofluorescence
Alexa Fluor 488 Donkey anti rat	Thermo Fisher Scientific, A-21208	1:200	Immunofluorescence
Alexa Fluor 488 Donkey anti goat	Thermo Fisher Scientific, A-11055	1:200	Immunofluorescence
Alexa Fluor 568 Donkey anti goat	Thermo Fisher Scientific, A11057	1:200	Immunofluorescence
Alexa Fluor 633 Donkey anti rabbit	Thermo Fisher Scientific, A-32795	1:200	Immunofluorescence
Alexa Fluor 647 Donkey anti rabbit	Thermo Fisher Scientific, A-21245	1:200	Immunofluorescence

Supplementary Figure 1

A

Wild-Type Mouse Islets							
	TrkA	***TrkB.FL***	***TrkB.T1***	***TrkB.T2***	***TrkC.FL***	***TrkC.T1***	***β-actin***
**ΔCt (*β-actin*)**	**9.67 ± 0.23**	**8.29 ± 0.31**	**3.20 ± 0.25**	**9.64 ± 0.21**	**13.99 ± 1.01**	**12.97 ± 0.86**	**-**
**Ct**	**29.03 ± 0.12**	**28.37 ± 0.27**	**23.29 ± 0.30**	**29.83 ± 0.19**	**32.86 ± 0.73**	**31.82 ± 0.26**	**19.59 ± 0.23**

B

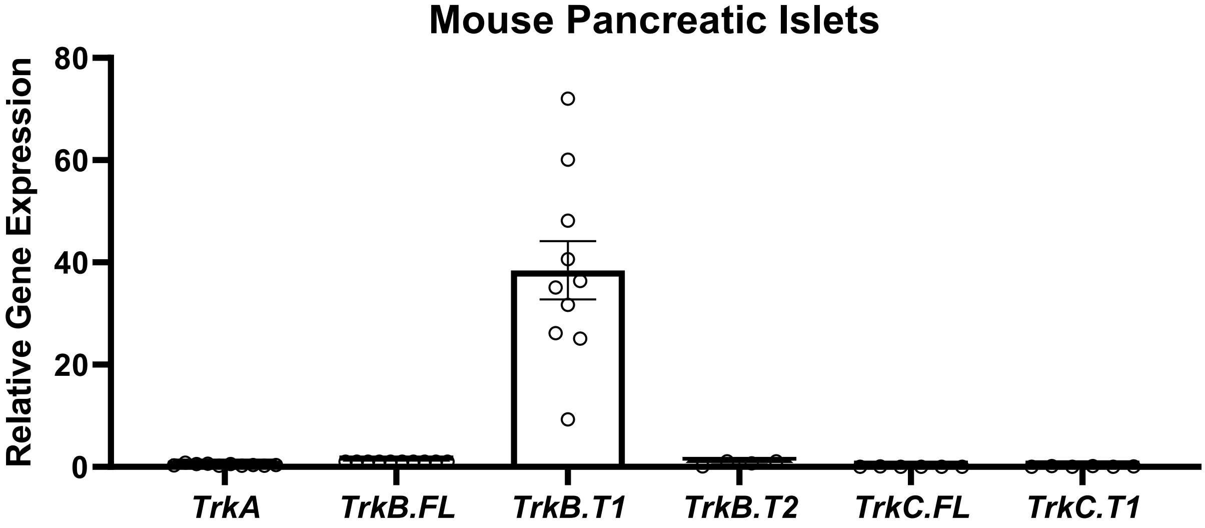

C

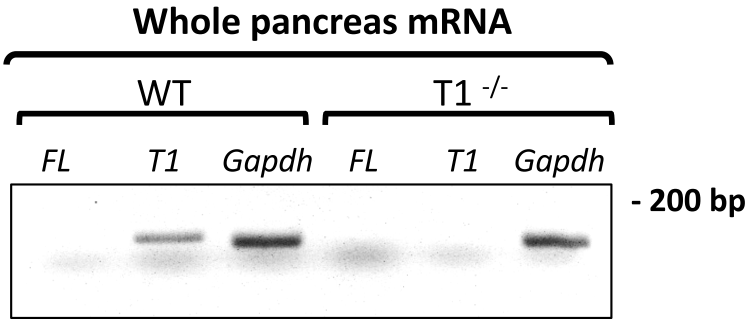

Supplementary Figure 1

TrkB.T1 is the most abundantly expressed Trk receptor isoform in mouse pancreatic islets. (A) Results of qRT-PCR analysis of *TrkA*, *TrkB* and *TrkC* receptor isoforms from isolated pancreatic islets expressed in ΔCt relative to *β-actin*. Ct values are also shown. (B) *Trk* mRNA expression in mouse islets based on data shown in (A); Relative fold of ΔCt of *TrkB.FL* and *β-actin* is set as 1. (C) Agarose gel of *TrkB.FL* (FL) and *TrkB.T1* (T1) real time PCR products from whole WT mouse pancreas lysates. Pancreas from TrkB.T1KO (T1 -/-) mice was used as control. Data represent mean ± S.E.M. Source data are provided as a Source Data file.

Supplementary Figure 2

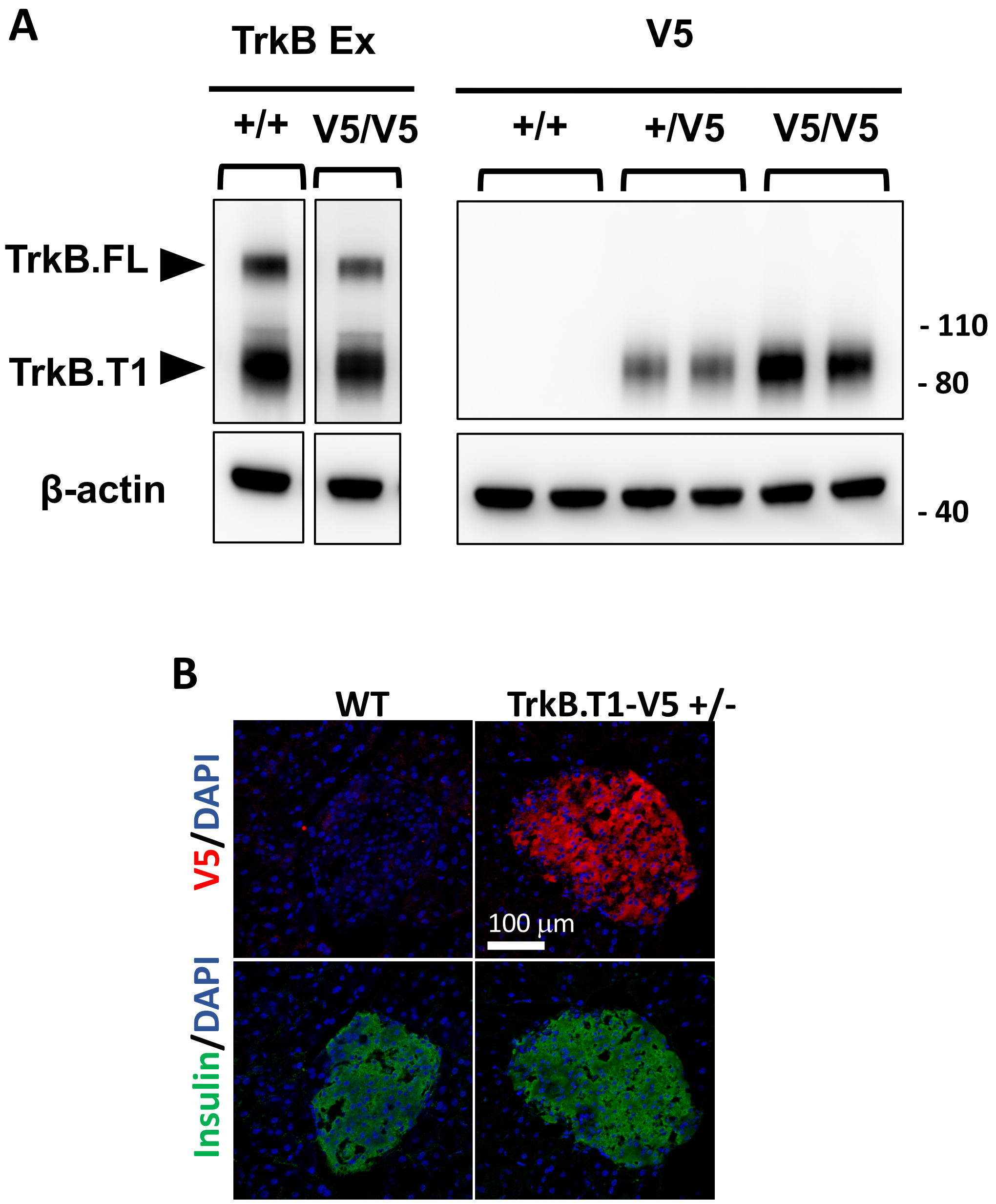

Supplementary Figure 2

V5-tagged TrkB.T1 mimics endogenous TrkB.T1 expression. (A) Western blot analysis of brain lysates from a control (+/+) and a homozygous (V5/V5) or heterozygous (+/V5) V5-tagged TrkB.T1 mouse using an antibody recognizing the extracellular domain of TrkB (TrkB Ex; left panels) or an anti-V5 antibody (right panels). β-actin was used as a loading control. (B) Immunofluorescence localization of V5-tagged TrkB.T1 in pancreatic slices. Note that the V5 signal (red) is detectable only in the endocrine pancreas (islets). WT pancreas was used as negative control. Tissues were co-stained with an anti-insulin antibody (green) and DAPI (blue). Source data are provided as a Source Data file.

Supplementary Figure 3

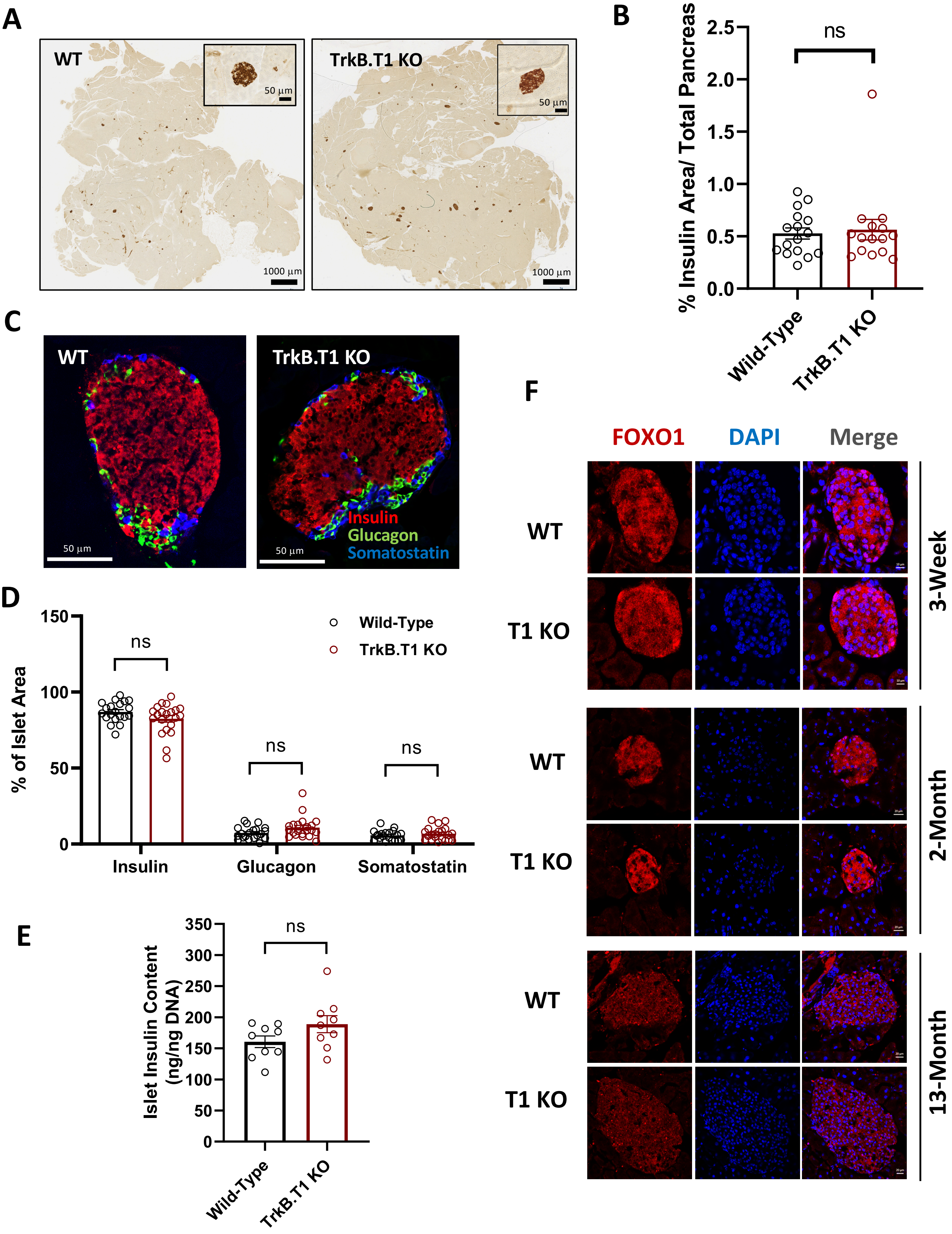

Supplementary Figure 3

TrkB.T1 deletion does not alter pancreatic islet development. (A) Immunoperoxidase insulin staining of Wild Type (WT) and TrkB.T1 deficient (KO) mouse pancreata. Note the overall similar size and distribution of insulin-stained (brown) islets; inset shows a typical islet. (B); Quantification of insulin-stained area as in (A) from WT and TrkB.T1 KO mouse pancreata, n=4 mice for both groups. Ratios of islet/total pancreas area were obtained by an automated procedure as described in methods. (C-D) Immunofluorescent staining of mouse islets with antibodies against insulin, glucagon and somatostatin (C) was used to determine the qualitative and quantitative (D) islet composition of WT and TrkB.T1 KO pancreata WT, n=20 islets; TrkB.T1 KO n=21 islets from 3 mice of each genotype. (E) Quantification of total insulin content from isolated WT and TrkB.T1 KO mouse islets relative to DNA content. n=9 mice for each group using 30 islets from each mouse. (F) Expression analysis of the transcription factor FoxO1 in 3-week (scale bar =10 µm), 2-month and 13-month (both scale bar =20 µm) old wild type (WT) and TrkB.T1 KO (T1 KO) mice, n=3 mice of each group. Note the similar gradual decrease of FoxO1 immunostaining during aging in both WT and TrkB.T1 mutant islets. Data represent mean ± S.E.M.. Source data are provided as a Source Data file.

Supplementary Figure 4

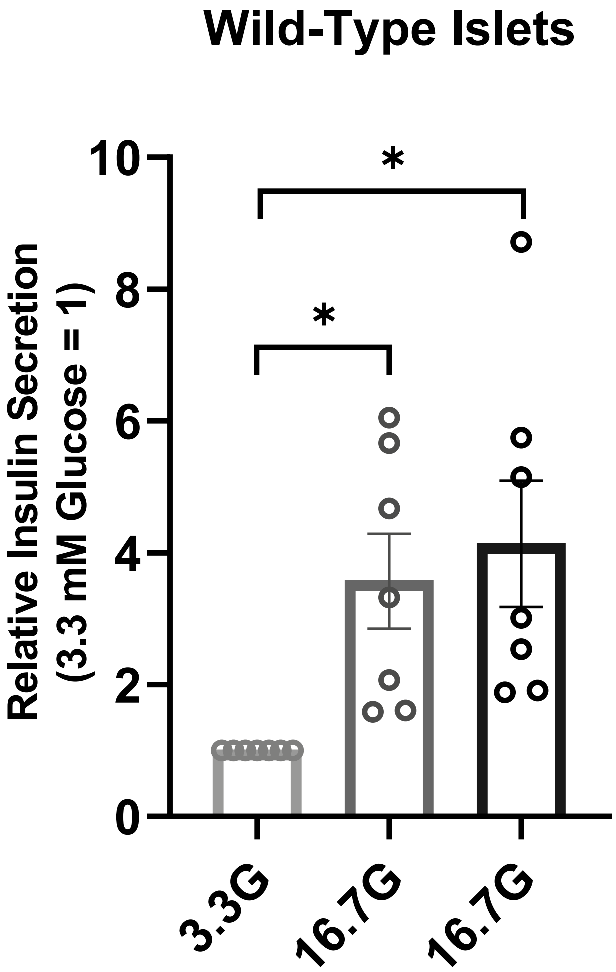

Supplementary Figure 4

Repeated exposure of mouse islets to high glucose levels does not increase insulin secretion. Insulin production of WT mouse islets incubated with 3.3mM glucose (3.3G) for 25 min, followed by two 25 min-stimulation periods with 16.7 mM glucose (16.7G) separated by a 25 min-wash (in 3.3 G). Data represent mean ± S.E.M. of 7 independent experiments. One-way repeated ANOVA *$p < 0.05$, followed by Tukey's test versus 3.3G-treated phase. Source data are provided as a Source Data file.

Supplementary Figure 5

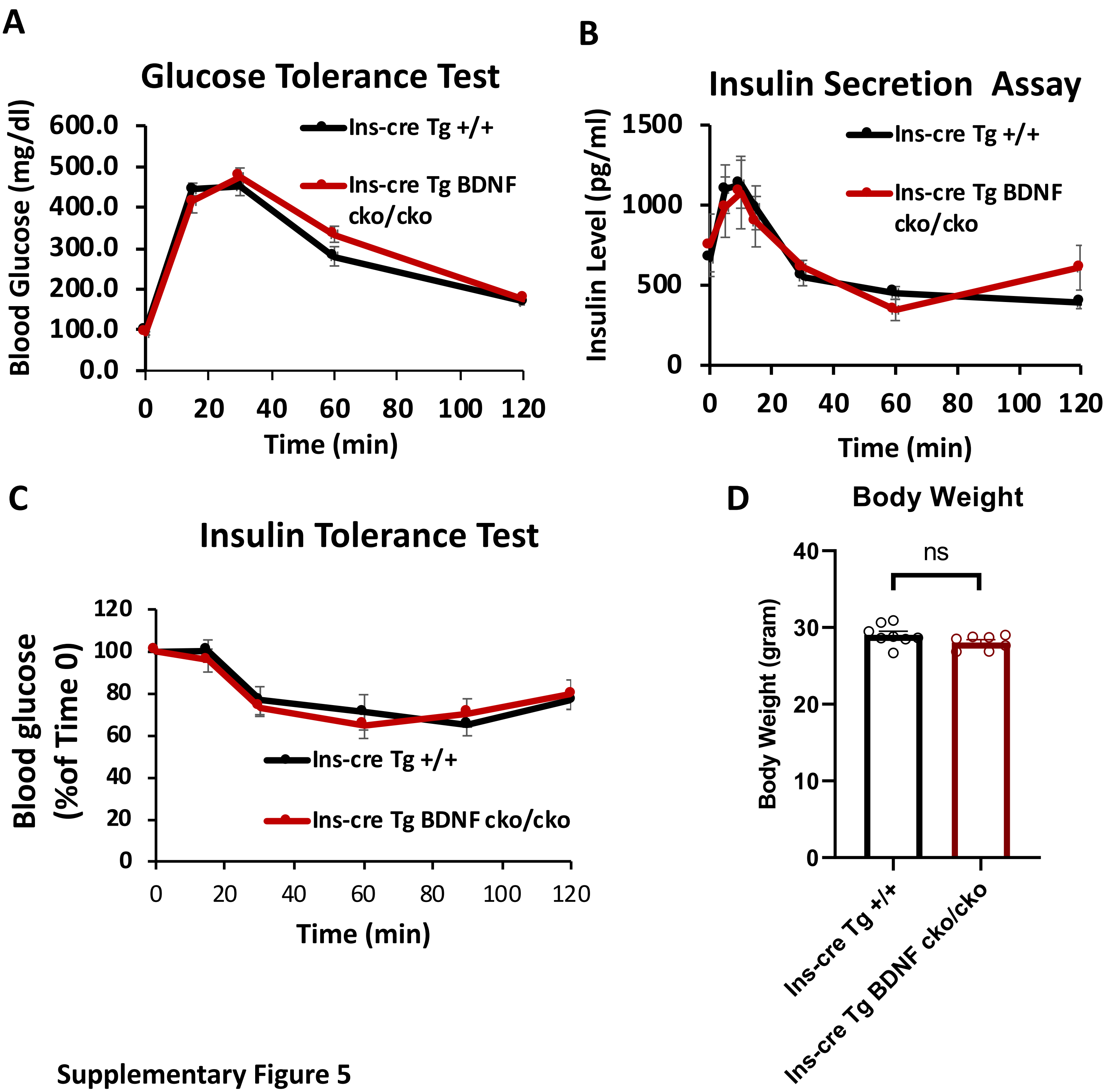

Supplementary Figure 5

Conditional deletion of BDNF in mouse pancreatic β-cells does not alter mouse responses to a glucose load. (A, B) Ins-cre Tg +/+ mice and Ins-cre Tg BDNF cko/cko mice show similar glucose tolerance (A) and glucose-induced insulin secretion (B) during an oral glucose tolerance test (2g/kg of glucose); Ins-cre Tg +/+, n=17 mice, Ins-cre Tg BDNF cko/cko, n=7 mice. (C); Insulin tolerance test show no differences between genotypes; Ins-cre Tg +/+ n=10 mice, Ins-cre Tg BDNF cko/cko n=7 mice. (D); Mutant and control mice have similar body weight at the time of testing. Ins-cre Tg +/+ n=8 mice, Ins-cre Tg BDNF cko/cko n=7 mice. Data represent mean ± S.E.M. Source data are provided as a Source Data file.

Supplementary Figure 6

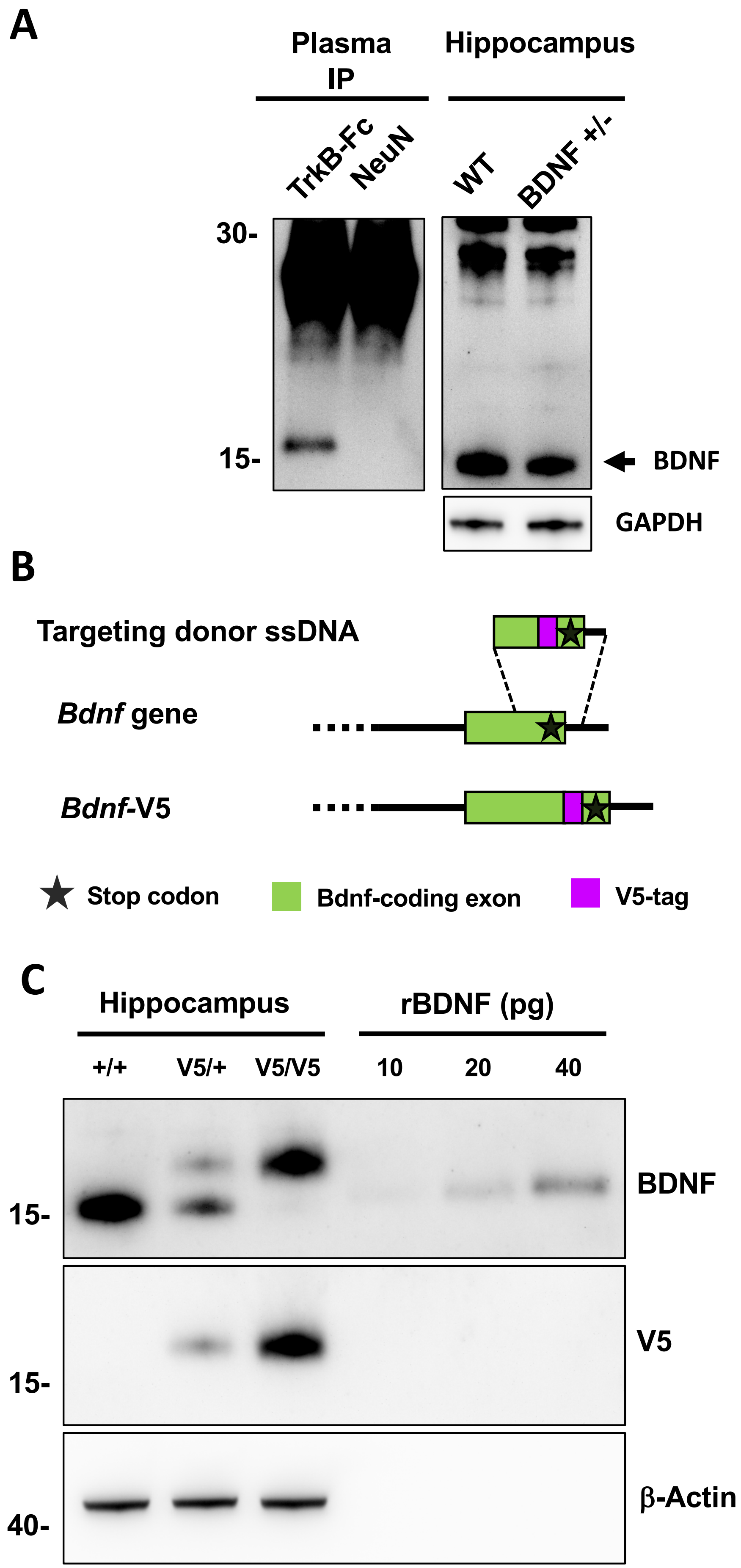

Supplementary Figure 6
BDNF is present in mouse plasma and knock-in of the V5 tag in the BDNF locus. (A) Detection of BDNF in mouse plasma; plasma from pooled blood of 6 mice was divided in 2 identical samples and immunoprecipitated with TrkB-Fc or an unrelated mouse monoclonal antibody (NeuN) before western blot analysis with a BDNF-specific monoclonal antibody. Hippocampal lysates from WT and BDNF+/- mice were used as controls. GAPDH was used as loading control. (B) Schematic diagram showing the strategy used to tag the BDNF gene with a V5 epitope to study its expression in mouse tissues. (C) Western blot analysis of hippocampal lysates from control (+/+), heterozygous (V5/+) and homozygous BDNF-V5 mice immunostained with a BDNF- (top panel) or a V5-specific antibody (middle panel). Note that the BDNF antibody recognizes two bands of different molecular weight in the BDNF-V5 heterozygous hippocampus consistent with the addition of 14 aa of the V5 tag to BDNF. Recombinant BDNF at different concentrations was used as positive control (top panel) and β-actin as control for loading (bottom panel). Source data are provided as a Source Data file.

Supplementary Figure 7

A

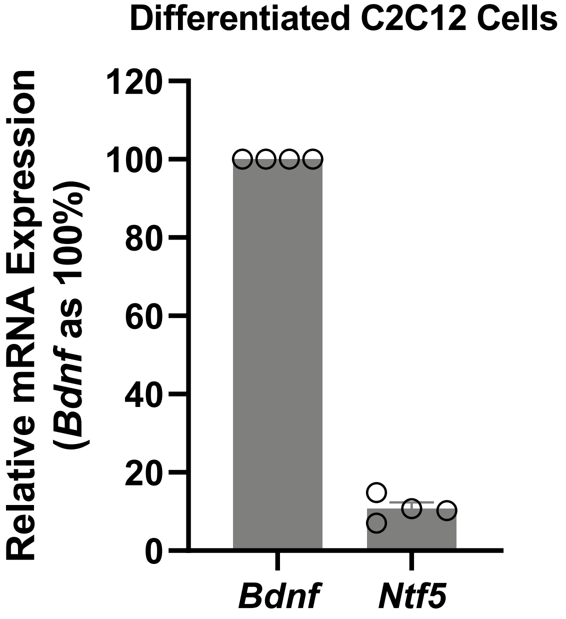

B

	Differentiated C2C12 cells		
	Bdnf	***Ntf5***	***Gapdh***
**ΔCt (*Gapdh*)**	8.46 ± 0.17	11.74 ± 0.09	-
**Ct**	23.02 ± 0.08	26.29 ± 0.16	14.56 ± 0.10

Supplementary Figure 7

***Bdnf* mRNA is the main TrkB receptor ligand expressed by differentiated C2C12 cells.** (A) *Bdnf* and *Ntf5* mRNA expression in differentiated C2C12 cells based on data shown in (B); Relative fold of ΔCt of *Bdnf* and *Gapdh* was set as 100%. (B) Results of qRT-PCR analysis of *Bdnf* and *Ntf5* expressed in ΔCt relative to *Gapdh*. Ct values are also shown. Data were expressed as mean ± S.E.M. from 4 independently differentiated samples. Source data are provided as a Source Data file.

Supplementary Figure 8

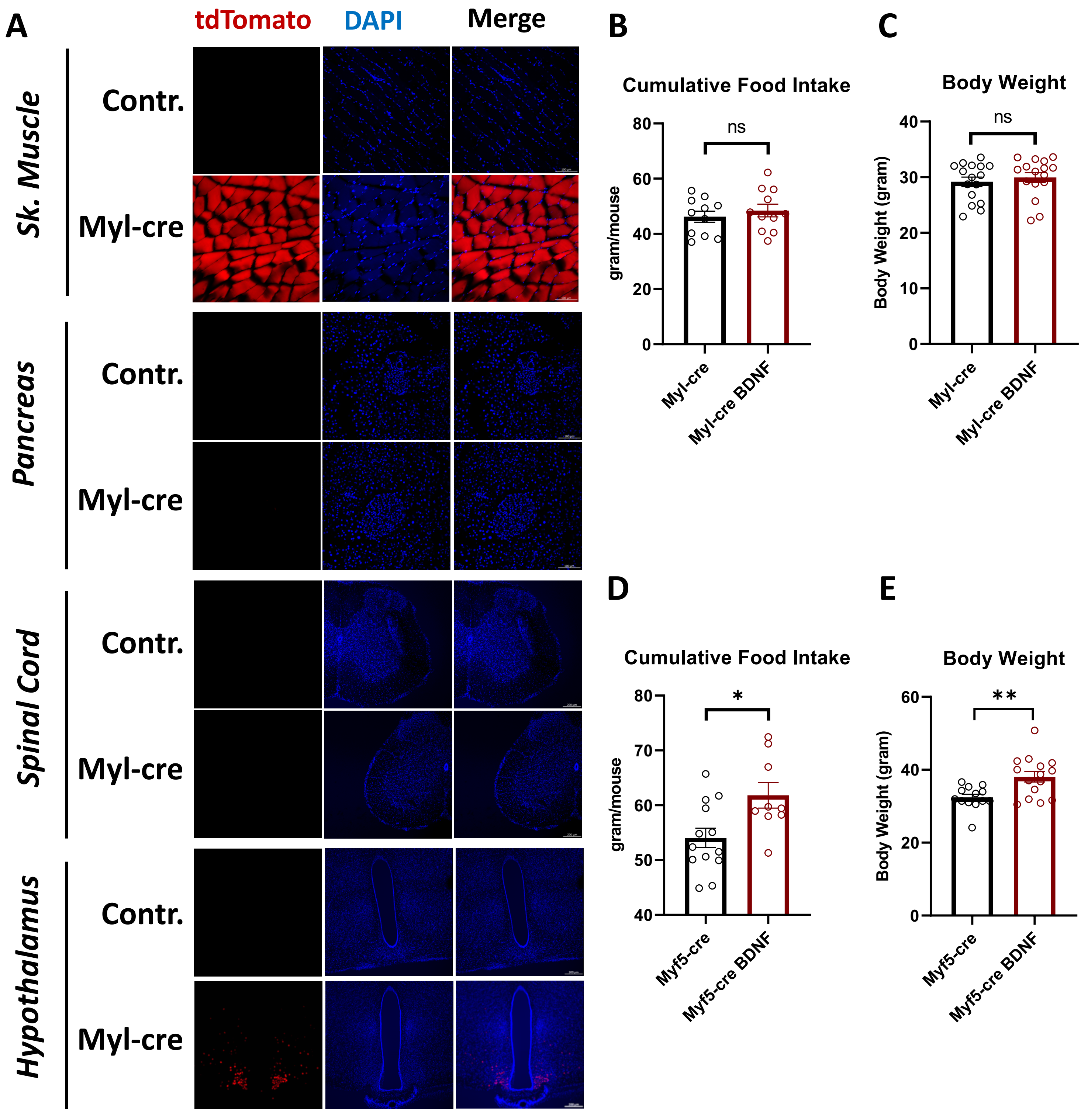

Supplementary Figure 8

Cre activity under the myosin light polypeptide 1 (Myl1) promoter is highly specific to skeletal muscle and Myl1-cre deletion of BDNF does not influence mouse food intake and body weight. (A); Crossing of the myosin light polypeptide 1-cre (Myl1-cre) transgenic mouse strain to the Rosa26-tdTomato reporter mouse shows strong specific red fluorescence in skeletal muscle. Staining of sections from the lumbar spinal cord, pancreas and hypothalamus showed an overall lack of signal. Only a few cells in the ventral hypothalamus appear to express the tdTomato but do not appear to overlap with the reported expression of BDNF (http://mouse.brain-map.org/gene/show/11850). (B-C); Mice with conditional deletion of BDNF in skeletal muscle (Myl-cre BDNF) do not show changes in food intake (B; n=11 mice for both genotypes) or body weight (C; n=16 mice for both genotypes), compared to control (Myl-cre) mice. Cumulative food intake was measured over a period of 10 days in singly housed mice, and mouse weight was measured at 3 months of age. (D-E); Mice with conditional deletion of BDNF with the skeletal muscle-specific Myf5-cre transgene show significant changes in food intake (D; Myf5-cre Tg+/+, n=13; Myf5-cre Tg cko/cko, n=9), or body weight (E; Myf5-cre Tg+/+, n=13; Myf5-cre Tg cko/cko, n=15). Cumulative food intake was measured over a period of 10 days in singly housed mice, and mouse weight was measured at 3 months of age. Student's t-test, *$p < 0.05$, **$p < 0.01$. Data were expressed as mean ± S.E.M. Source data are provided as a Source Data file.

Supplementary Figure 9

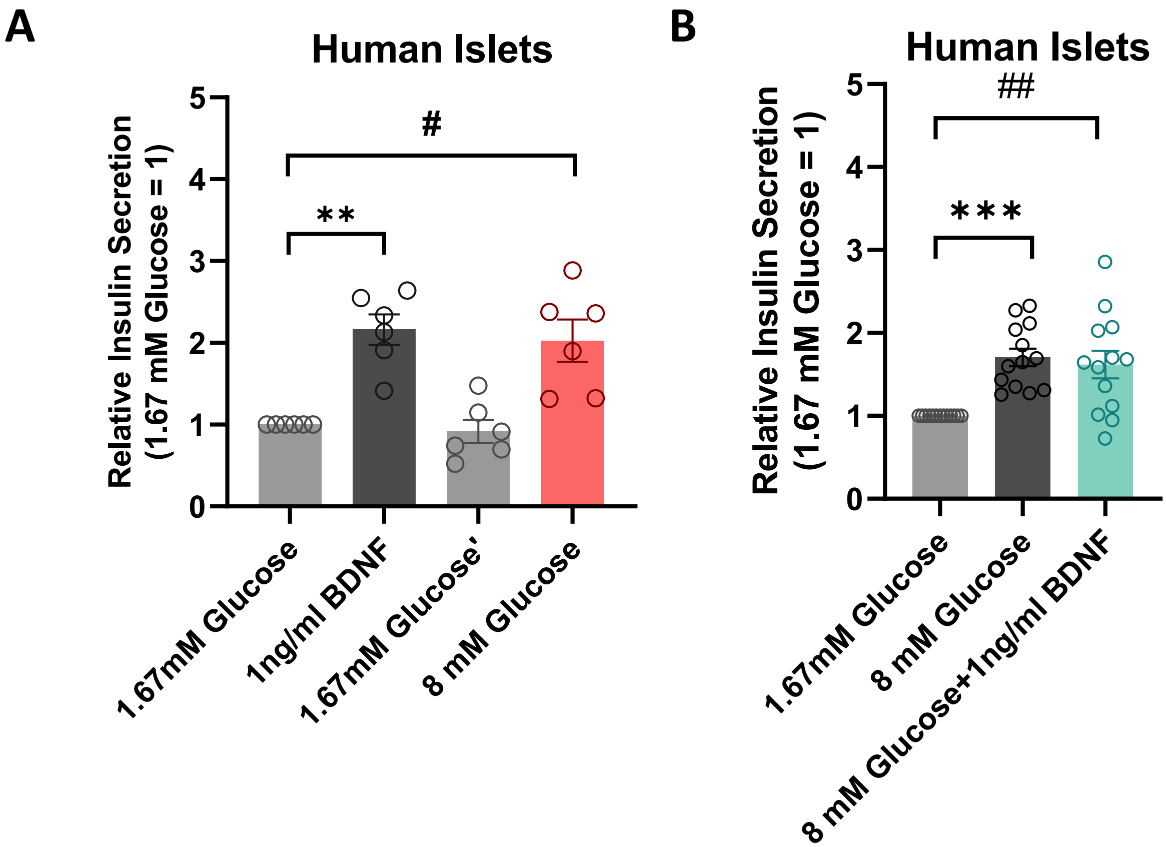

Supplementary Figure 9

BDNF induction of insulin release from human islets does not synergize with high glucose. Priming of human islets with BDNF does not increase the amount of insulin secretion induced by glucose alone (A) and no synergistic effect was observed under the condition of BDNF with high concentration of glucose (B). Quantification of changes in insulin secretion levels relative to islets treated with 1.67 mM glucose. Conditions are as indicated in the panels and include islets incubations for 25 minutes with 1 ng/ml BDNF (A) or 8 mM glucose solution (B), followed by a 25-minute wash in 1.67 mM glucose and the last treatment with an 8 mM glucose solution (A) or 8 mM glucose plus 1 ng/ml BDNF solution (B). One-way repeated ANOVA $^{**}p < 0.01$, $^{***}p < 0.001$ followed by Tukey's test between the first 1.67 mM glucose phase and the second phase; $^{\#}p < 0.05$, $^{\#\#}p < 0.01$ followed by Tukey's test between the first 1.67 mM glucose-treated phase and the last phase; A, n=6; B, n=13 independent experiments. Data were expressed as mean ± S.E.M. Source data are provided as a Source Data file.
